# Cremation and Urn-Burial

**Published:** 1889-11-30

**Authors:** 


### Cremation and Urn-Burial.

In a sanitary sense there can be no manner of doubt of the superiority of cremation* over burial. It is stated with some truth that in about six years there is little or nothing left of a human body buried in the earth, provided it has not been protected by lead or some other impervious structure. Still, in the progress of decay, matter more or less injurious to the living must be evolved. Gases, the product of putrefaction, must be transmitted to and evolved from the superlayer of earth, and salts of various kinds must be dissolved in and conveyed by water and drainage in different directions. Moreover, the time of decay of a body when placed in the earth must depend in a great measure on the peculiar earth in which it is laid. A body would not decay in clay soil, for instance, nearly so soon as in a gravel or porous soil. That the sanitary evils of burial have been recognised is sufficiently evident by the present custom of extra-urban cemeteries. Now, the principal objections against cremation are, first, the sentimental one. Persons do not like the idea of the rapid destruction of the bodies of their relatives. But after death the buried body returns to its elements by a process of slow decay and putrefaction. The burnt body also returns to its elements, although by a more rapid process. The ultimate result is, however, the same in either case. Next, there is what may be termed the religious objection. Many persons are foolish enough to think that the resurrection of the body would be interfered with by cremation. But to this Canon Liddon replied: "The resurrection of a body from its ashes is not a greater miracle than the resurrection of an unburnt body; each must be purely miraculous." Next, there is the medico-legal objection, some persons opining that crime would be facilitated by cremation. But as Dr. Cameron, M.P., said: "The medico-legal objection has no weight whatever; and for this reason, that persons are allowed to bury their dead without the smallest precaution." Moreover, the Cremation Society has established stringent rules under this head, rendering the medico-legal objection altogether untenable.

Mr. Robinson, in the book under notice, considers the subject from another point of view—the æsthetic one. He describes how graveyards have been desecrated—the half-decayed bones and mould shovelled away, and the site made available for some other purpose. Several instances of this have occurred in London. The cemetery of the future should not only be a garden, in the best sense of the word, but the most beautiful and best cared for of all gardens. In old Roman cemeteries may yet be seen tombs with the urns within them, in as good order as when placed there 2,000 years ago. For the ancient Romans, although they believed in immortality, still believed in burning the bodies of their dead. Numerous pictures of tombs and urns are given, demonstrating how ornamental both may be made. In connection with this part of the subject are considered the opportunities which urn-burial would afford for depositing the inoffensive remains of the dead in our churches.

As an appendix to Mr. Robinson's book is attached the rules of the Cremation Society of England. Any person may become a member, under certain conditions. The arrangements for cremating a body at St. John's, Woking, are complete. The charge for cremation is £6. In this connection it may be mentioned that the cost of burning a Hindoo body in Bombay, where wood is dear, does not amount to more than rs. 3 ans. 8, or, say, seven shillings. But the burning is effected in the open air without any costly apparatus. Cremation must be made cheaper than burial in this country for it to become popular.

* "Cremation and Urn-Burial; or, the Cemeteries of the Future." By W. Robinson. Cassell and Co. 1889.

